# False Discovery Rates in PET and CT Studies with Texture Features: A Systematic Review

**DOI:** 10.1371/journal.pone.0124165

**Published:** 2015-05-04

**Authors:** Anastasia Chalkidou, Michael J. O’Doherty, Paul K. Marsden

**Affiliations:** Division of Imaging Sciences and Biomedical Engineering, Kings College London 4th Floor, Lambeth Wing, St. Thomas Hospital, SE1 7EH, London, United Kingdom; Stanford University Medical Center, UNITED STATES

## Abstract

**Purpose:**

A number of recent publications have proposed that a family of image-derived indices, called texture features, can predict clinical outcome in patients with cancer. However, the investigation of multiple indices on a single data set can lead to significant inflation of type-I errors. We report a systematic review of the type-I error inflation in such studies and review the evidence regarding associations between patient outcome and texture features derived from positron emission tomography (PET) or computed tomography (CT) images.

**Methods:**

For study identification PubMed and Scopus were searched (1/2000–9/2013) using combinations of the keywords texture, prognostic, predictive and cancer. Studies were divided into three categories according to the sources of the type-I error inflation and the use or not of an independent validation dataset. For each study, the true type-I error probability and the adjusted level of significance were estimated using the optimum cut-off approach correction, and the Benjamini-Hochberg method. To demonstrate explicitly the variable selection bias in these studies, we re-analyzed data from one of the published studies, but using 100 random variables substituted for the original image-derived indices. The significance of the random variables as potential predictors of outcome was examined using the analysis methods used in the identified studies.

**Results:**

Fifteen studies were identified. After applying appropriate statistical corrections, an average type-I error probability of 76% (range: 34–99%) was estimated with the majority of published results not reaching statistical significance. Only 3/15 studies used a validation dataset. For the 100 random variables examined, 10% proved to be significant predictors of survival when subjected to ROC and multiple hypothesis testing analysis.

**Conclusions:**

We found insufficient evidence to support a relationship between PET or CT texture features and patient survival. Further fit for purpose validation of these image-derived biomarkers should be supported by appropriate biological and statistical evidence before their association with patient outcome is investigated in prospective studies.

## Introduction

This is an exciting era for imaging biomarkers. Fast computing and state of the art software has facilitated the collection and analysis of large amounts of data, while the development of data mining techniques enables researchers to test a large number of hypotheses simultaneously. The utilization of imaging biomarkers is evolving from qualitative interpretation to more sophisticated quantitative analysis with the use of various image-based metrics. In the same way that gene array and molecular biomarkers led to the analysis of complex interaction models, similarly a number of image analysis algorithms and image-derived features are promising to unravel complex tumour biology by overcoming the limitations inherent in invasive tissue sampling techniques.

The most commonly used metrics currently applied to positron emission tomography (PET) images are the standardised uptake value (SUV) derived indices. These include SUVmax, the voxel with the maximum activity concentration in the tumour; SUVmean, calculated by averaging the activity concentration in all voxels inside a tumour volume; SUVpeak, calculated by averaging the voxel values inside a small region of interest centred on the SUVmax; the metabolically active tumour volume (MTV), and total lesion glycolysis (TLG), which is the product of MTV and the SUVmean. These metrics are all closely associated with tumour burden and metabolism and whilst there is ongoing debate about the best index to use in a given clinical situation, there is a large literature documenting the links between these indices and clinical outcomes. The index most commonly derived from computed tomography (CT) images is a measurement of tumour volume, often characterised by measurements of the tumour diameter using methods described by, for example, the RECIST criteria [1]. Recently, the application of image classification techniques to PET and CT images has resulted in a new family of indices [2,3], known as texture features, that have been used to characterise tumour heterogeneity.

Cancer heterogeneity is a phenomenon associated with clonal branch evolution (genetic variability) and regional differences in the tumour microenvironment (non-genetic variability) [4,5]. In brief, it has been proposed that most neoplasms arise from a single cancer cell, and that the inherent genomic instability of the cancer cells leads to mutations and the acquisition of genetic variability within the original clone [6]. The subclone selection is based on evolutionary factors governed by Darwinian principles that arise from interactions between the tumour microenvironment and the cancer cell properties [4,7]. An example of the role tumour microenvironment plays is tumour hypoxia, which leads to the selection of aggressive subclones exhibiting high metastatic potential and leading to poor patient outcome [8,9]. Mapping heterogeneity across spatial scales, from the cellular level to medical imaging, requires not only objective reproducible metrics for imaging features but also a theoretical construct that bridges those scales [10]. Although several researchers attempted to establish a general model of texture description [11,12], it is generally recognized that no general mathematical model of texture based only on statistical data-driven methods can be used to solve every image analysis problem [10,13]. There are some critical aspects to consider when designing texture operators to model tumour heterogeneity [13]. For 3-D texture feature analysis in particular the main aspects to consider are the scale in which heterogeneity is being examined (from μm for microscopy to cm for PET), the voxel size since this is the elementary building block of a given texture class, the slice thickness, whether the 3-D lattice is anisotropic or isotropic, the noise in the data [13]. The majority of texture features that have been used in PET and CT medical imaging to date fall into one the following three categories: a) first-order features derived from statistical moments of the image intensity histogram, b) second-order features derived from the gray level co-occurrence matrix, and c) higher order features derived from analysis of the neighbourhood gray-tone difference matrix or gray level size-zone matrices [13].

We have however identified a number of serious deficiencies in the way that the majority of investigations into these new image-derived indices, and their potential for use as imaging biomarkers, are conducted. Firstly, the methodology for such investigations typically includes the determination of the optimum value from a continuous distribution of values of the image-derived index, such that the patient population is divided into high and low risk groups. Multiple cut-off values are tested in order to find an optimum value (i.e. the value that has the most statistically significant relationship with outcome) using receiver operating characteristic (ROC) analysis. This will be referred to as the ‘optimum cut-off approach’, or according to Altman et al [14] ‘the minimum p-value approach’. The use of optimum cut-offs is not new in the field of imaging biomarkers. Berghmans et al [15] have previously identified, in a systematic review and meta-analysis, that, in 61% of the studies included, the choice of the SUV threshold between patients with high survival and low survival was based on the optimum cut-off.

There are a number of problems with the optimum cut-off approach. Hilsenbeck et al [16] demonstrated that as the number of possible cut-offs examined increases, so does the likelihood of erroneously obtaining a statistically significant result. Additionally, as different datasets have different optimal cut-offs it is not possible to replicate the optimal cut-off in different studies, thus making the quantification of the prognostic value impossible. Lastly, there is a tendency to overestimate the effect size [14,17], in this case the association between texture features and outcome. Although there are methods for the correction of type-I errors (the error of rejecting a null hypothesis when it is actually true, commonly referred to as a false positive), the overestimation of the effect size cannot be calculated or corrected for, and ultimately this will lead to claiming a factor as of prognostic relevance, when in fact it does not have any influence on prognosis.

Secondly, whilst previously, only a handful of indices would be tested when searching for potential new imaging biomarkers, now numerous image-derived indices can increase this number by 10-fold, leading to multiple hypothesis testing. The effects of the optimum cut-off approach and multiple hypothesis testing, outlined above and examined in detail below, are well known and documented in other fields, for example in tissue biomarker analysis. Their combination during the analysis of a single study in the field of imaging biomarkers heightens the potential type-I error inflation and so warrants caution.

In addition to the above statistical considerations, the use of texture features in predicting response is based on the hypothesis that they characterize tumour heterogeneity and hence contain complementary information to that provided by indices like SUV or tumour volume. To date, evidence for this association has not been reported, however several studies have shown that most PET texture features are highly correlated both with each other and with tumour volume [18–22]. This collinearity between texture features can lead to the phenomenon known as ‘bouncing betas’ [23], this relates to the instability of the regression coefficient weights in a multivariate model when multicollinearity exists between variables and small changes in the data lead to very different regression coefficients.

A number of contributing factors that in general add to the probability of a research finding being false are listed in [24]. These are: small sample size, great number and lesser selection of tested relationships, and great flexibility in design, definitions, outcomes and analytical modes. These factors can easily be recognised in most imaging biomarker studies but get amplified in cases where multiple image-derived indices with no pre-specified analytical model are used.

In the light of the issues outlined above, the aim of the study presented here was, firstly, to investigate the extent of the inflation of the type-I error rate in PET and CT imaging biomarker studies using texture features conducted with the methodology outlined above, and secondly, to examine the evidence supporting an association between PET and CT texture features and patient outcome in these studies following the application of appropriate statistical corrections. A systematic review of studies investigating the use of PET or CT image-derived texture features to predict patient outcomes was performed. In addition, in order to demonstrate explicitly the variable selection bias in these studies, 100 random variables were generated, and their significance as potential predictors of outcome was examined on a previously published dataset, following the same methodology that was used in the original study.

## Materials and Methods

### Study identification and selection

Publications satisfying the following criteria were eligible for consideration:

1. Inclusion of patients with any cancer type
2. Investigation of the relationship between different texture features extracted from PET or CT images and clinical outcome
3. Publication as a full paper in a peer-reviewed scientific journal.

### Search methods

A search of studies published in PubMed and Scopus (2000–2013) was performed. The most recent search was done in September 2013. Both subject headings and free text were used for the search. The search was performed with a combination of terms related to PET, CT and texture, with no language restrictions and limited to human studies. The full electronic search strategy for Pubmed is listed in S1 Table.

### Data extraction and management

For each study the following were extracted on two different occasions by one researcher (AC):

1. Number of univariate analyses performed per study (i.e. how many hypotheses were tested per study)
2. Method employed for obtaining a cut-off with prognostic power (i.e. ROC analysis, mean or other)
3. Did the authors perform any adjustment of the p-value in order to control the increase in type-I error probability resulting from a) multiple hypothesis testing or b) the use of the optimum cut-off approach
4. Presence of ad-hoc analysis (was a pre-specified hypothesis tested)
5. Presence and use of a validation dataset to confirm results
6. Presence of cross-correlation analysis (i.e. did authors perform a cross correlation analysis to examine for possible dependencies amongst the variables tested)

RevMan version 5.2 was used for data collection and management [25].

### Type-I error rate estimation and adjustment of significance level

The studies included in the review were divided into three categories according to the sources of the type-I error inflation present:

- Studies with multiple hypothesis testing only
- Studies employing both multiple hypothesis testing and the optimum cut-off approach
- Studies with multiple hypothesis testing, with or without the optimum cut-off approach, but with validation analysis

In order to determine the true type-I error probability, corrections were applied as follows:

For studies in category A the Benjamini-Hochberg correction for multiple hypothesis testing (which is considered more powerful and less conservative than the Bonferroni procedure [26]) was applied. In this method the variables are ranked according to their p-values in increasing order. For a significance level p = 0.05, those that satisfy the relationship $p_{\left(k\right)}\leq \frac{k}{m}\times 0.05$(m equals to the number of comparisons and k equals to the p-value) are considered statistically significant.

For studies in category B the adjustment was done in two steps. Firstly, a correction to the minimal p-values obtained from the optimum cut-off approach was performed using the formula developed by Altman et al [14], and then the Benjamini-Hochberg procedure was applied.

For studies in category C no corrections were made.

Regarding the correction for the optimum cut-off approach applied in category B studies, as described in [14], if P_min_ represents the minimum p-value of the log-rank statistic obtained from each study, the corrected p-value (for 0.0001<P_min_<0.1), P_cor_, is obtained as follows:

$$P_{cor}=-1.63\times P_{\min}\times \left(1+2.35\times \ln\;P_{\min}\right)\;for\;\varepsilon =10\%$$ (1)

Where ε is the proportion of values from the tails of the continuous variable distribution that is excluded during the ROC analysis (10% from each end of the distribution), leaving the rest of the distribution (80%) to be considered for possible cut-offs. In most cases performing an ROC analysis with a statistical software package such as SPSS (SPSS Inc.) will include all values of the distribution, thus making the selection of ε = 10% less conservative and allowing more significance after the correction. The P_cor_ calculated with formula 1 was then compared with the adjusted significance level in order to achieve an overall type-I error probability of 0.05 based on the Benjamini-Hochberg procedure. A spreadsheet that implements the Benjamini and Hochberg method for calculating the corrected significance level when multiple hypotheses are tested was used [27].

### Demonstration of selection bias using random variables

Survival data were extracted from Ganeshan et al [28] for 21 patients with oesophageal cancer, and overall survival was used as an end point. The relationship between 100 random variables and overall survival was assessed. The random variables were generated in Excel using the normal random number generator formula below:

NORMSINV(RAND())×m+(SD)

Values for the mean (m = 0.016) and standard deviation (SD = 0.02) were selected to match those of the coarseness texture feature in order to be unrelated to the survival dataset under analysis whilst still retaining the statistical properties of the texture feature [29]. To obtain a more accurate percentage estimate of the number of false predictors expected, the analysis was repeated, using 100 random variables. An optimal cut-off for the random variables was calculated from ROC curves based on the minimum p-value approach. Kaplan-Meier curves were used to investigate the impact of the random variables on patient survival and a nonparametric log-rank test was used to calculate the differences between the two survival curves. In a similar way to previous publications, no sample size calculation, correction for multiple hypothesis testing or correction for use of the optimum cut-off approach was performed. Any p-value of less than 0.05 was considered significant. The statistical software IBM SPSS version 21 was used.

## Results

### Study identification and selection

**Fig 1:**
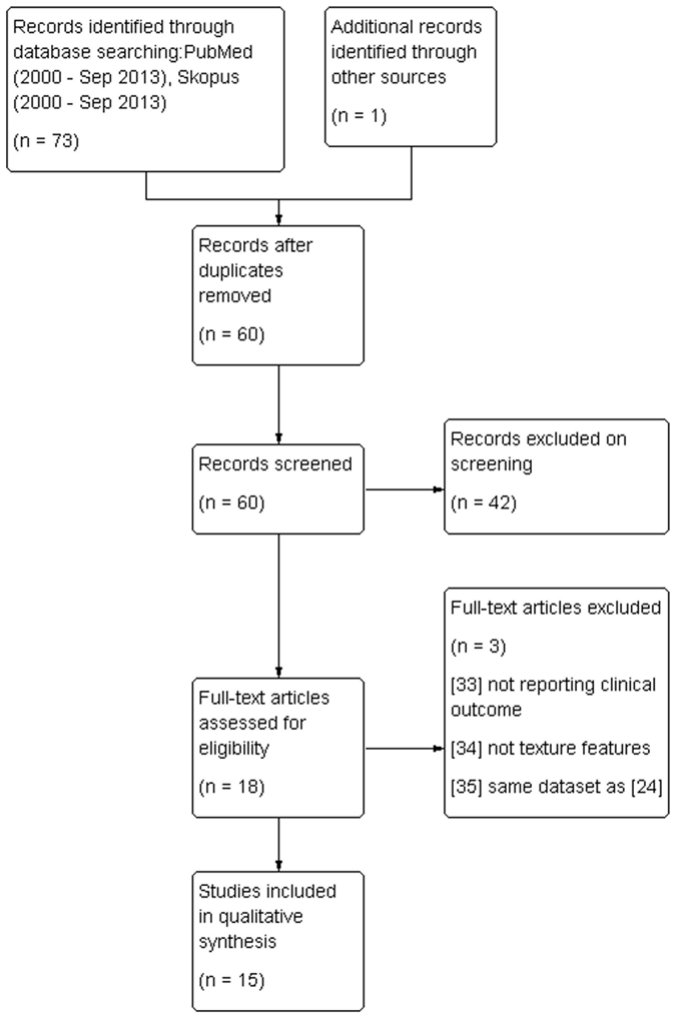
Study flow diagram according to PRISMA guidelines.

The original search in Pubmed and Scopus databases identified 73 articles. After removing duplicates, 60 abstracts were screened according to the evaluation criteria, and 17 in total were selected to be read in full as potentially eligible. In addition, one further study [19] was identified through an alternative source. Fifteen studies [19,28–41] were selected for inclusion in the review, while three studies were excluded with reasons [42–44]. Fig 1 describes the study flow diagram according to the PRISMA guidelines for reporting systematic reviews [45].

### Study characteristics

The selected studies were published between 2009 and 2013. The mean number of patients analysed per study was 44 (range 12–72). The mean number of hypotheses tested per study was 38 (range 8–102). Studies covered a range of cancer sites. Their characteristics are summarised in Tables 1 and 2. Technical information of texture features implementation in CT studies and PET studies are summarised in S2 and S3 Table.

**Table 1:** Statistical characteristics of the selected studies divided in three categories: A) Studies with multiple hypotheses testing only, B) studies employing both multiple hypothesis testing and the optimum cut-off approach and C) studies with multiple hypothesis testing, with or without the optimum cut-off approach, but with validation analysis.

Category	Study	Multivariate analysis included volume	Optimum cut-off	Type I error adjustment	Validation dataset	cross correlation reported	Sample size	Hypotheses tested
A	Willaime [19]	Not applicable	No/Mean	No	No	Yes	12	68
	El Naqa [31]	NI*	Not clear	No	No	No	14/9	19
	Tixier [33]	NI	Not clear	No	No	Yes	41	54
	Yip [41]	No	No/Median	Yes^#^	No	No	36	90
B	Miles [30]	No	Yes	No	No	No	48	10
	Goh [32]	No	Yes	No	No	No	39	24
	Cook [29]	No	Yes	No	No	Yes	53	30
	Ganeshan [28]	No	Yes	No	No	Yes	21	15
	Ganeshan [34]	No	Yes	No	No	No	54	8
	Ng [36]	No	Yes	No	No	Yes	55	25
	Zhang [40]	Yes	Yes	No	No	No	72	40
	Cheng [39]	Yes	Yes	No	No	Yes	70	59^‡^
C	Vaidya [35]	Yes	No	No	LOOCV^†^	No	27	102
	Win [37]	No	Yes	No	Yes	No	66	12
	Ravanelli [38]	No	No/Median	No	LOOCV	No	53	16

* No information provided

^#^For multiple hypotheses tested

^†^Leave one out cross validation

^‡^ Number is a conservative approximation due to the difficulty establishing the exact number of hypotheses tested

**Table 2:** General characteristics of selected studies.

Study	Cancer type	Modality*	Tracer	Feature	Relationship with good outcome	Timing
Miles [30]	Colorectal	CT	NA	Uniformity (2.0/2.5)^‡^	>0.907	baseline
El Naqa [31]	Cervical, H&N	PET	FDG	Model/Various	NA	pre/post
Ng [36]	Colorectal	CT	NA	Entropy, Uniformity (1.0)	>7.89, <0.005	baseline
Goh [32]	Renal mets	CT	NA	Uniformity (2.5)	>-2%	delta
Tixier [33]	Esophageal	PET	FDG	Local features and Regional features	No information	baseline
Cook [29]	NSCLC	PET	FDG	Coarseness	low	baseline
Ganeshan [28]	Esophageal	CT	NA	Uniformity (2.5)	>0.84	baseline
Ganeshan [34]	NSCLC	CT	NA	Uniformity (2.5)	>0.62	baseline
Vaidya [35]	NSCLC	PET/CT	FDG	Model	NA	baseline
Win [37]	NSCLC	PET/CT	FDG	Entropy (1.5/2.5)	>1.23	baseline
Willaime [19]	Breast	PET	FLT	FBP^†^: No statistical significance	FBP: not applicable	FBP: pre/post
	Breast	PET	FLT	OSEM†: CV,AUC-CSH, Entropy, Complexity	OSEM: low, high, high, low	OSEM: baseline
Zhang [40]	HNSCC	CT	NA	Skewness	low	baseline
Cheng [39]	HNSCC	PET	FDG	Uniformity (4 bins)	>0.138	baseline
Yip [41]	Esophageal	CT	NA	Uniformity (1.5/2.0/2.5), Entropy (1.5/2.0)	>0.007, <7.35	post-Tx
Ravanelli [38]	NSCLC	CT	NA	Uniformity and grey level (U*GL, sigma value = 4)	II-III tertiles	baseline

*Modality texture analysis was performed on

^†^Filtered back projection, Iterative reconstruction

^‡^Numbers represent different filtration levels

### Statistical analysis

Four [19,31,33,41], eight [28–30,32,34,36,39,40] and three studies [35,37,38] were assigned to categories A, B and C respectively (Table 1).

**Fig 2:**
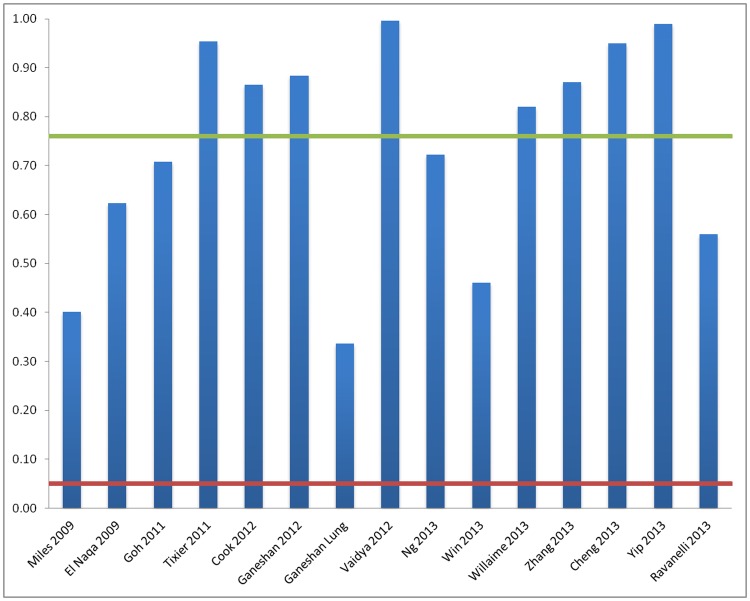
Probability of a false positive result based on number of hypotheses tested per study (blue columns) for all study categories. 5% type-I error probability = red line, average type-I error probability (76%) over all studies = green line (Note—additional inflation of the type-I error probability due to the use of the optimum cut-off approach is not included here).

**Fig 3:**
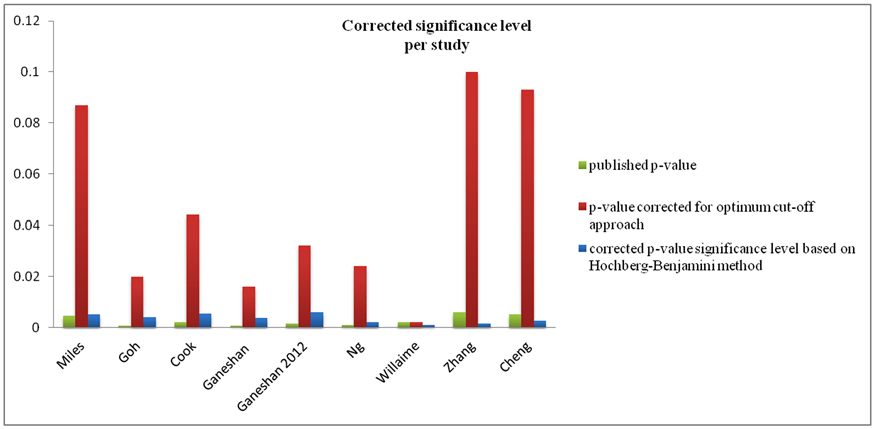
Studies from categories A and B after adjustments for optimum cut-off approach and/or multiple hypotheses testing. Green column demonstrates the smallest published p-value per study, the red the P_cor_ for the optimum cut-off approach, and the blue the corrected statistical significance level based on Hochberg-Benjamini method. For a study to have a statistical significant result the red column value should be smaller than the green blue which is not the case for any of them. For study [19] the green and red column are identical as investigators did not use the optimum cut-off approach. Studies [31,33] and [41] were excluded as they did not provide a summary of their p-values for correction, and had adjusted the results for multiple hypotheses, respectively.

Fig 2 shows, for studies from categories A and B, the corrected type-I error probability for each study and the average type-I error probability over all studies (76%) based on the number of hypotheses tested. Fig 3 shows the result for the smallest published p-value quoted in each study after correcting for the use of the optimum cut-off approach and adjusting the significance level using the Benjamini-Hochberg procedure. For B category studies the additional type-I error source due to the optimum cut-off method is not included in Fig 2 but is accounted during the adjustment of the significance level in Fig 3. None of the studies in categories A and B for which it was feasible to apply the corrections retained statistically significant results after the corrections had been applied. Studies [31,33] were excluded because they did not provide a summary of their p-values for correction and study [41] was excluded because results were already adjusted for multiple hypotheses. For category C study [38] no associations between the various texture features and survival were claimed in the publication, while in [35] no associations between texture features and patient outcome were claimed with the exception of the intensity-volume histogram (IVH) (a surrogate for tumour volume). In [37] an association between the CT texture feature entropy and survival was claimed but no association was established between PET texture features and survival.

**Fig 4:**
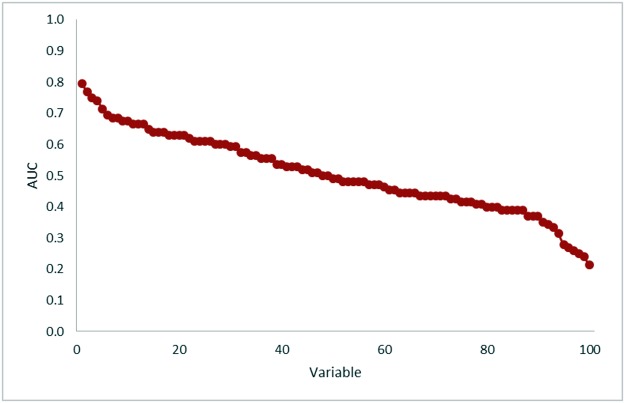
Area under the curve (AUC) values from receiver operating characteristic (ROC) analysis of 100 random variables. The variables are ordered by decreasing AUC values.

**Fig 5:**
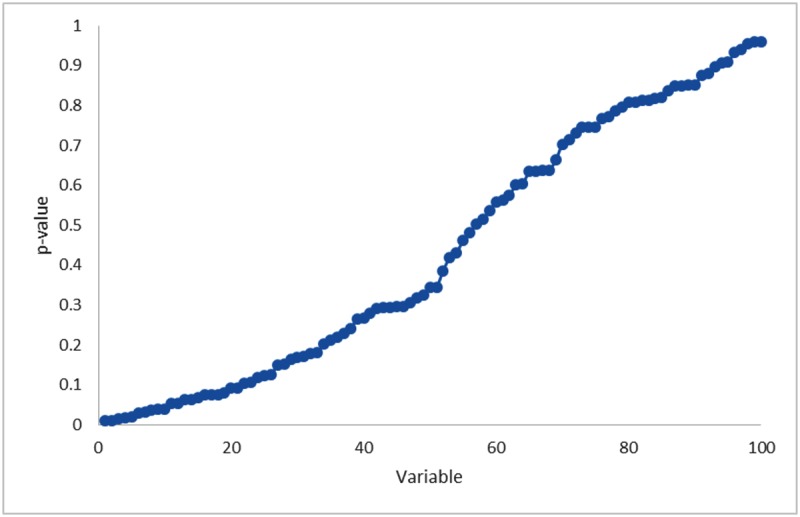
Statistical significance of Kaplan-Meier analysis for 100 random variables using the optimum cut-off approach. The variables are ordered by increasing p-values. Overall 10% of the random variables are statistically significant predictors of survival.

The minimum and maximum AUC achieved with the random variables were 0.213 and 0.796, respectively (Fig 4). In comparison with the texture features investigated in the studies retrieved from the systematic review, the random variable analysis achieved higher AUCs than uniformity in [28,30,32,34], energy in [31], or busyness in [29]. Despite there being no real relationships between the 100 random variables and survival, using the methodology typically employed in the published studies, in 10% of the variables the choice of an optimum cut-off appeared to have prognostic power in Kaplan Meier survival analysis (Fig 5). The AUC values for these random variables with prognostic power are reported in Table 3.

**Table 3:** AUC values after ROC analysis for the generated 10 random variables.

Variable	AUC
random1	.796
random2	.769
random3	.750
random4	.741
random5	.713
random6	.694
random7	.685
random8	.684
random9	.676
random10	.675

**Fig 6:**
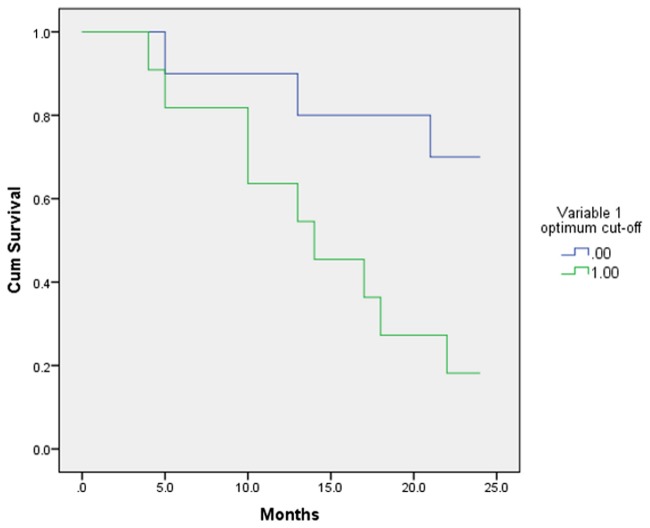
Kaplan Meier curves based on optimum cut-off value for the random variable 1.

**Fig 7:**
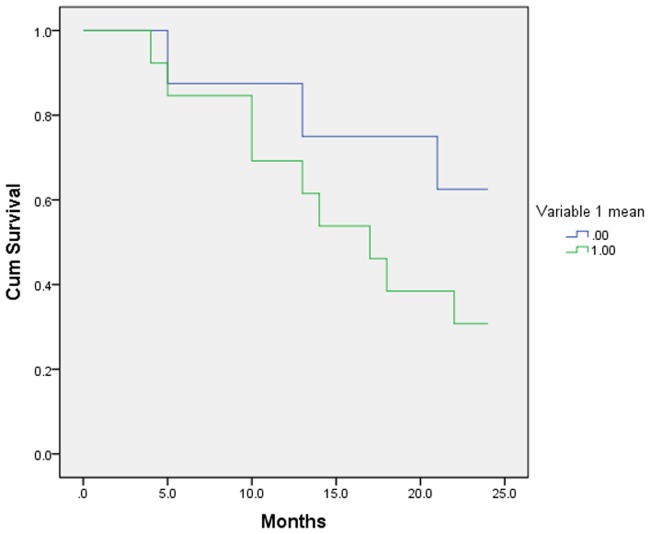
Kaplan Meier curves based on mean value for the random variable 1.

As an example, the Kaplan-Meier curves results are demonstrated for one variable (random variable 1) in Figs 6 and 7. Survival was higher for patients with a random variable 1 cut-off <0.01556 (group 1) with mean survival 20.7 months (CI: 16.86–24.53 months), and lower for patients with a random variable 1 cut-off >0.01556 (group 2) and mean survival 14.63 months (CI: 10.65–18.61 months), based on Kaplan-Meier analysis and the log-rank test (p = 0.020, Fig 6). In order to compare the results when a single cut-off was used instead of multiple cut-offs (ROC analysis) the mean value of random variable 1 (as defined by the surviving vs. non surviving groups) was also used to calculate the Kaplan-Meier curves. When the mean value was used, no difference in survival of the two groups was noted (p = 0.178, Fig 7).

## Discussion

It is common practice to retrospectively analyse patient datasets to provide a proof of concept that may motivate further exploration of a biomarker. This step is followed by the design of a prospective study with the aim of definitively testing the hypothesis generated. The process of testing multiple cut-offs during ROC analysis and multiple image-derived metrics, which are often not independent of each other, is likely to lead to positive results. However, these results will not be reproducible and the actual size of the effect will be overestimated and falsely associated with clinical end points.

This is confirmed from our systematic review findings. As predicted from the theory, out of 15 studies analysed we were unable to find any two studies that identified the same texture feature and/or cut-off value as of prognostic significance, even when the same modality and cancer type were analysed. The most alarming finding was that in some cases the same texture feature was linked to both positive and negative patient outcomes in different studies. For example, while in [28] higher baseline uniformity was associated with good prognosis in oesophageal cancer, in [36] patients needed to have lower baseline uniformity to achieve good prognosis in colorectal cancer. Additionally the results of [28] in oesophageal cancer regarding the prognostic values of baseline uniformity were not confirmed in [41].

The term biomarker refers to a measurable indicator of some biological state or condition. Texture features have been introduced as imaging biomarkers with the assumption that they are an index of the degree of tumour heterogeneity. It is widely accepted that biological tumour heterogeneity is associated with poor prognosis in cancer patients as it can contribute to treatment failure and drug resistance, and this has important consequences for personalized-medicine [4,46,47]. Based on this assumption, tumours with higher biological heterogeneity are expected to be associated with poorer survival, and even if colorectal and oesophageal cancer are two different cancer types it is still expected that heterogeneity would have the same effect on patient prognosis. An equivalent scenario with an established index would be, for example, that a large tumour volume indicated a poor prognosis in some cancer types but a good one in others. Finally, it may be that texture features behave differently for different cancer types because they do not measure tumour heterogeneity but some other biological property. A characteristic example of discordance between radiological and biological heterogeneity is the comparison between a histopathology diagnosis of bronchiolo-alveolar carcinoma (BAC) and the radiological finding of ground glass opacity (GGO) on high-resolution CT. The appearance of small lung adenocarcinomas in CT can vary consisting of solid and GGO component [48]. In CT a nodule featuring 100% GGO will be considered as of increased radiological heterogeneity in comparison with a nodule that consists of 100% solid component. It has been shown that in patients with small solitary lung adenocarcinomas the % BAC component in histology correlated well with the % GGO component on CT, and that the prognosis was better if the nodule had a high % of GGO [49]. Based on the new histopathologic classification of adenocarcinoma [50] the term BAC has been discontinued and substituted by the term non-invasive adenocarcinoma. As a result tumours with a higher % of GGO component, therefore a high percentage of non-invasive carcinoma and low biological heterogeneity, will have an excellent prognosis [51]. On the contrary tumours with a higher % of solid component, therefore a higher percentage of invasive adenocarcinoma and higher biological heterogeneity, will have a worse prognosis [51]. Consequently, for radiological heterogeneity to accurately reflect biological heterogeneity the underlying mechanism of biological heterogeneity needs to be taken into account when designing these imaging features.

As part of our analysis, we generated 100 random variables and used the same process that was used in the published studies to test their prognostic value. Out of 100 random variables tested, 10% proved to be significant predictors of survival when the cut-off value was chosen using the optimum cut-off approach. As a result, we were able to identify a significant but clinically implausible association between survival and our variables because of the over-inflation of the type-I error caused by combining the optimum cut-off approach and multiple hypothesis testing statistical analysis.

The retrospective analysis of data sets with texture features has not managed, in some cases, to reproduce well established associations between certain variables and patient outcome, reflecting the limitations of retrospective analysis and of employing small, heterogeneous cohorts of patients. For example, in [29] no association was found between stage and survival analysis, while in [39] no association was found between HPV status or stage and disease-specific survival. Small sample sizes not only increase the type-I error rate but also reduce the probability of detecting a true difference between groups, where one exists (type-II error). To be able to generate accurate estimates of the impact of the depended variables an adequate number of events per variable is needed. It has been proposed that for linear models, such as multiple regression, a minimum of 10 to 15 observations per predictor variable will produce reasonably stable estimates [52,53]. In the field of imaging biomarkers, the lack of interpretations of the image-derived indices in terms of meaningful biological end points, makes this approach susceptible to error. These associations should be specified during the design of the study, as it is tempting to construct biologically plausible reasons for observed subgroup effects after having observed them [54].

Only 3/15 of the studies included in the review [35,39,40] added tumour volume into the multivariate analysis. Collinearity between PET texture features and tumour volume will influence the regression coefficients estimation and will increase the type-I error as a function of the indices correlation value [55]. For example, in [56] it was demonstrated that the inclusion of tumours with volumes of less than 45cm^3^ biases tracer uptake heterogeneity studies toward statistically significant differences even when none are present. As a result the use of univariate and multivariate analysis, adopted in the vast majority of texture feature studies, is problematic and highlights the need for validation analysis.

The necessity for multiple comparison correction has been a long standing debate, especially when performing an exploratory analysis. Ultimately the only confirmation of the validity of the results is by verifying the outcome of the exploratory analysis in a validation dataset. From our review, we identified only 3 studies that included validation of their results [35,37,38]. In [35] and [38], after cross validation analysis no association between texture features and patient outcome was identified. According to the principles of validation analysis, an independent dataset is required to confirm the results of a previous study, without changing any of the original analysis parameters [57,58]. In [37] a different CT texture feature and optimal cut-off were selected as significant between the original study that analysed the same dataset by Ganeshan et al. in 2012 [34] (Uniformity, cut-off = 0.6236) and the subsequent validation study [37] that included the same training dataset (Entropy, cut-off = 1.233), questioning the prospective nature of the analysis. To facilitate the development of best practices for the analysis of imaging data involving new image-derived biomarkers and algorithms, these need to be compared and validated on datasets that are large and diverse [59]. Because data of adequate quality are sparse, it is important to support data sharing activities such as the Cancer Imaging Archive and encourage investigators to share the raw imaging data after publication[59].

Texture features are susceptible to various sources of variability such as different acquisition modes and reconstruction parameters [35,37,38], and different levels of discretisation [35]. Different reconstruction algorithms have different noise properties and this will affect the texture properties of the resulting images. In [60] from 50 texture features examined only one, first-order entropy, showed low variability due to the reconstruction method but was still susceptible to the image grid size and SUV scaling. In [57,58] no prognostic information from texture features was provided when FBP reconstruction was used, but significant associations were identified with OSEM in the same dataset. Recently, two further studies investigated the test-retest and interobserver reproducibility of FDG-PET [61] and CT [62] texture features. Useful commentaries on the misconceptions, possible sources of variability and limitations of texture features analysis are provided in [63,64].

The present study has some limitations. Firstly, study authors were not contacted to provide additional data or verify the extracted study characteristics. However, regarding additional data provision there were only 2 cases [31,33] for which we couldn’t identify information in the published manuscript for estimating the type-I error and both these were studies without a validation dataset. Secondly, the data extraction was performed by one investigator only. However, the data extraction list did not include any subjective information (e.g. methodological quality items) that could have been subject to debate, and the process was repeated on two separate occasions.

The field of imaging biomarkers is continuously expanding. Validation studies of imaging biomarkers are methodologically challenging, time consuming and expensive. Resources for conducting these studies are not unlimited, and ethical considerations exist regarding testing hypotheses on patients without robust data. Furthermore, the long-term follow up required for providing confirmation of the value of a biomarker will take years to complete. As a result, priorities in the selection of markers to be investigated further must be based on robust evidence. In an era where the lack of reproducibility in research findings has become one of the most significant problems [65], emerging trends in the field of imaging biomarkers should be carefully scrutinised for the validity of their results. There are recent examples in the field of image-derived biomarkers where cancer stratification models were developed by combining clinical, imaging and gene expression data using large multicentre datasets, with multiple external validation sets and from various cancer sites to reduce the risk of type-I errors [66].

Various publications have outlined the theoretical and practical limitations of using regression analysis for the development of patient outcome prediction models [52,67,68]. In general, the following basic steps will help reduce false discoveries and ensure that the model provides not only statistically significant but also clinically relevant results: a) variable reproducibility assessment, b) cross-correlation analysis, c) inclusion of clinically important variables (such as disease stage and treatment received), d) an adequate event rates (at least >10–15 per variable tested), e) use of an external validation cohort ensuring that the same texture feature and cut-off are tested.

## Conclusion

After appropriate statistical corrections for the probability of type-I errors and a review of the published results, we found insufficient evidence, much of it conflicting, to support a relationship between PET or CT texture features and patient outcome. Fit for purpose validation of image-derived biomarkers should be supported by appropriate biological and statistical evidence before prospective studies of their association with patient outcome are performed.

## Supporting Information

S1 PRISMA Checklist PRISMA checklist. (DOCX)

S1 Table Electronic search strategy for Medline Ovid interface. (DOCX)

S2 Table Technical information of texture features implementation in CT studies. (DOCX)

S3 Table Technical information of texture features implementation in PET studies. (DOCX)

## References

[1] Wahl RL, Jacene H, Kasamon Y, Lodge MA (2009) From RECIST to PERCIST: Evolving Considerations for PET response criteria in solid tumors. J Nucl Med 50 Suppl 1: 122S–150S. 10.2967/jnumed.108.057307 19403881 PMC2755245

[2] Haralick RM, Shanmugam K, Dinstein IH (1973) Textural Features for Image Classification. Systems, Man and Cybernetics, IEEE Transactions on SMC-3: 610–621.

[3] Amadasun M, King R (1989) Textural features corresponding to textural properties. Systems, Man and Cybernetics, IEEE Transactions on 19: 1264–1274.

[4] Gerlinger M, Rowan AJ, Horswell S, Larkin J, Endesfelder D, Gronroos E, et al (2012) Intratumor heterogeneity and branched evolution revealed by multiregion sequencing. The New England journal of medicine 366: 883–892. 10.1056/NEJMoa1113205 22397650 PMC4878653

[5] Schwarz RF, Ng CK, Cooke SL, Newman S, Temple J, Piskorz AM, et al (2015) Spatial and temporal heterogeneity in high-grade serous ovarian cancer: a phylogenetic analysis. PLoS Med 12: e1001789 10.1371/journal.pmed.1001789 25710373 PMC4339382

[6] Nowell PC (1976) The clonal evolution of tumor cell populations. Science 194: 23–28. 959840 10.1126/science.959840

[7] Fernebro J, Engellau J, Persson A, Rydholm A, Nilbert M (2007) Standardizing evaluation of sarcoma proliferation- higher Ki-67 expression in the tumor periphery than the center. APMIS 115: 707–712. 17550378 10.1111/j.1600-0463.2007.apm_650.x

[8] Brizel DM, Scully SP, Harrelson JM, Layfield LJ, Bean JM, Prosnitz LR, et al (1996) Tumor oxygenation predicts for the likelihood of distant metastases in human soft tissue sarcoma. Cancer Res 56: 941–943. 8640781

[9] Brizel DM, Sibley GS, Prosnitz LR, Scher RL, Dewhirst MW (1997) Tumor hypoxia adversely affects the prognosis of carcinoma of the head and neck. Int J Radiat Oncol Biol Phys 38: 285–289. 9226314 10.1016/s0360-3016(97)00101-6

[10] Gatenby RA, Grove O, Gillies RJ (2013) Quantitative imaging in cancer evolution and ecology. Radiology 269: 8–15. 10.1148/radiol.13122697 24062559 PMC3781355

[11] Haralick RM (1979) Statistical and structural approaches to texture. Proceedings of the IEEE 67: 786–804.

[12] Julesz B (1981) Textons, the elements of texture perception, and their interactions. Nature 290: 91–97. 7207603 10.1038/290091a0

[13] Depeursinge A, Foncubierta-Rodriguez A, Van De Ville D, Müller H (2014) Three-dimensional solid texture analysis in biomedical imaging: Review and opportunities. Medical Image Analysis 18: 176–196. 10.1016/j.media.2013.10.005 24231667

[14] Altman DG, Lausen B, Sauerbrei W, Schumacher M (1994) Dangers of using "optimal" cutpoints in the evaluation of prognostic factors. J Natl Cancer Inst 86: 829–835. 8182763 10.1093/jnci/86.11.829

[15] Berghmans T, Dusart M, Paesmans M, Hossein-Foucher C, Buvat I, Castaigne C, et al (2008) Primary tumor standardized uptake value (SUVmax) measured on fluorodeoxyglucose positron emission tomography (FDG-PET) is of prognostic value for survival in non-small cell lung cancer (NSCLC): a systematic review and meta-analysis (MA) by the European Lung Cancer Working Party for the IASLC Lung Cancer Staging Project. J Thorac Oncol 3: 6–12. 10.1097/JTO.0b013e31815e6d6b 18166834

[16] Hilsenbeck SG, Clark GM, McGuire WL (1992) Why do so many prognostic factors fail to pan out? Breast cancer research and treatment 22: 197–206. 1391986 10.1007/BF01840833

[17] Hilsenbeck SG, Clark GM (1996) Practical p-value adjustment for optimally selected cutpoints. Statistics in medicine 15: 103–112. 8614741 10.1002/(SICI)1097-0258(19960115)15:1<103::AID-SIM156>3.0.CO;2-Y

[18] Bagci U, Yao J, Miller-Jaster K, Chen X, Mollura DJ (2013) Predicting future morphological changes of lesions from radiotracer uptake in 18F-FDG-PET images. PLoS One 8: e57105 10.1371/journal.pone.0057105 23431398 PMC3576352

[19] Willaime JM, Turkheimer FE, Kenny LM, Aboagye EO (2013) Quantification of intra-tumour cell proliferation heterogeneity using imaging descriptors of 18F fluorothymidine-positron emission tomography. Phys Med Biol 58: 187–203. 10.1088/0031-9155/58/2/187 23257054

[20] Siddique M GV, Marsden P, Taylor B, Frost M., Blake G, Cook G. Correlation between textural features of 18F-FDG PET in oesophageal cancer.; 2013 4 2013; Brighton United Kingdom Lippincott Williams and Wilkins.

[21] Huang B, Chan T, Kwong DL, Chan WK, Khong PL (2012) Nasopharyngeal carcinoma: investigation of intratumoral heterogeneity with FDG PET/CT. AJR American journal of roentgenology 199: 169–174. 10.2214/AJR.11.7336 22733909

[22] Orlhac F, Soussan M, Maisonobe JA, Garcia CA, Vanderlinden B, Buvat I (2014) Tumor texture analysis in 18F-FDG PET: relationships between texture parameters, histogram indices, standardized uptake values, metabolic volumes, and total lesion glycolysis. J Nucl Med 55: 414–422. 10.2967/jnumed.113.129858 24549286

[23] Kiers HL, Smilde A (2007) A comparison of various methods for multivariate regression with highly collinear variables. Statistical Methods and Applications 16: 193–228.

[24] Ioannidis JPA (2005) Why Most Published Research Findings Are False. PLoS Med 2: e124 16060722 10.1371/journal.pmed.0020124 PMC1182327

[25] The Nordic Cochrane Centre (2012) Review Manager (RevMan). In: The Cochrane Collaboration, editor. 5.2 ed Copenhagen.

[26] Benjamini Y, Hochberg Y (1995) Controlling the False Discovery Rate: A Practical and Powerful Approach to Multiple Testing. Journal of the Royal Statistical Society Series B (Methodological) 57: 289–300.

[27] Weinkauf M (2012) BenjaminiHochberg.xlsx 1.1 ed.

[28] Ganeshan B, Skogen K, Pressney I, Coutroubis D, Miles K (2012) Tumour heterogeneity in oesophageal cancer assessed by CT texture analysis: preliminary evidence of an association with tumour metabolism, stage, and survival. Clin Radiol 67: 157–164. 10.1016/j.crad.2011.08.012 21943720

[29] Cook GJ, Yip C, Siddique M, Goh V, Chicklore S, Roy A, et al (2013) Are pretreatment 18F-FDG PET tumor textural features in non-small cell lung cancer associated with response and survival after chemoradiotherapy? J Nucl Med 54: 19–26. 10.2967/jnumed.112.107375 23204495

[30] Miles KA, Ganeshan B, Griffiths MR, Young RC, Chatwin CR (2009) Colorectal cancer: texture analysis of portal phase hepatic CT images as a potential marker of survival. Radiology 250: 444–452. 10.1148/radiol.2502071879 19164695

[31] El Naqa I, Grigsby P, Apte A, Kidd E, Donnelly E, Khullar D, et al (2009) Exploring feature-based approaches in PET images for predicting cancer treatment outcomes. Pattern Recognit 42: 1162–1171. 20161266 10.1016/j.patcog.2008.08.011 PMC2701316

[32] Goh V, Ganeshan B, Nathan P, Juttla JK, Vinayan A, Miles KA (2011) Assessment of response to tyrosine kinase inhibitors in metastatic renal cell cancer: CT texture as a predictive biomarker. Radiology 261: 165–171. 10.1148/radiol.11110264 21813743

[33] Tixier F, Le Rest CC, Hatt M, Albarghach N, Pradier O, Metges JP, et al (2011) Intratumor Heterogeneity Characterized by Textural Features on Baseline 18F-FDG PET Images Predicts Response to Concomitant Radiochemotherapy in Esophageal Cancer. J Nucl Med 52: 369–378. 10.2967/jnumed.110.082404 21321270 PMC3789272

[34] Ganeshan B, Panayiotou E, Burnand K, Dizdarevic S, Miles K (2012) Tumour heterogeneity in non-small cell lung carcinoma assessed by CT texture analysis: a potential marker of survival. Eur Radiol 22: 796–802. 10.1007/s00330-011-2319-8 22086561

[35] Vaidya M, Creach KM, Frye J, Dehdashti F, Bradley JD, El Naqa I (2012) Combined PET/CT image characteristics for radiotherapy tumor response in lung cancer. Radiother Oncol 102: 239–245. 10.1016/j.radonc.2011.10.014 22098794

[36] Ng F, Ganeshan B, Kozarski R, Miles KA, Goh V (2013) Assessment of primary colorectal cancer heterogeneity by using whole-tumor texture analysis: contrast-enhanced CT texture as a biomarker of 5-year survival. Radiology 266: 177–184. 10.1148/radiol.12120254 23151829

[37] Win T, Miles KA, Janes SM, Ganeshan B, Shastry M, Endozo R, et al (2013) Tumor heterogeneity and permeability as measured on the CT component of PET/CT predict survival in patients with non-small cell lung cancer. Clin Cancer Res 19: 3591–3599. 10.1158/1078-0432.CCR-12-1307 23659970

[38] Ravanelli M, Farina D, Morassi M, Roca E, Cavalleri G, Tassi G, et al (2013) Texture analysis of advanced non-small cell lung cancer (NSCLC) on contrast-enhanced computed tomography: prediction of the response to the first-line chemotherapy. Eur Radiol 23: 3450–3455. 10.1007/s00330-013-2965-0 23835926

[39] Cheng NM, Fang YH, Chang JT, Huang CG, Tsan DL, Ng SH, et al (2013) Textural features of pretreatment 18F-FDG PET/CT images: prognostic significance in patients with advanced T-stage oropharyngeal squamous cell carcinoma. J Nucl Med 54: 1703–1709. 10.2967/jnumed.112.119289 24042030

[40] Zhang H, Graham CM, Elci O, Griswold ME, Zhang X, Khan MA, et al (2013) Locally advanced squamous cell carcinoma of the head and neck: CT texture and histogram analysis allow independent prediction of overall survival in patients treated with induction chemotherapy. Radiology 269: 801–809. 10.1148/radiol.13130110 23912620

[41] Yip C, Landau D, Kozarski R, Ganeshan B, Thomas R, Michaelidou A, et al (2014) Primary esophageal cancer: heterogeneity as potential prognostic biomarker in patients treated with definitive chemotherapy and radiation therapy. Radiology 270: 141–148. 10.1148/radiol.13122869 23985274

[42] Tan S, Kligerman S, Chen W, Lu M, Kim G, Feigenberg S, et al (2013) Spatial-temporal [(1)(8)F]FDG-PET features for predicting pathologic response of esophageal cancer to neoadjuvant chemoradiation therapy. Int J Radiat Oncol Biol Phys 85: 1375–1382. 10.1016/j.ijrobp.2012.10.017 23219566 PMC3606641

[43] Gensure RH, Foran DJ, Lee VM, Gendel VM, Jabbour SK, Carpizo DR, et al (2012) Evaluation of Hepatic Tumor Response to Yttrium-90 Radioembolization Therapy Using Texture Signatures Generated from Contrast-enhanced CT Images. Academic Radiology 19: 1201–1207. 10.1016/j.acra.2012.04.015 22841288 PMC3438382

[44] Hatt M, Tixier F, Cheze Le Rest C, Pradier O, Visvikis D (2013) Robustness of intratumour (1)(8)F-FDG PET uptake heterogeneity quantification for therapy response prediction in oesophageal carcinoma. Eur J Nucl Med Mol Imaging 40: 1662–1671. 10.1007/s00259-013-2486-8 23857457

[45] Liberati A, Altman DG, Tetzlaff J, Mulrow C, Gotzsche PC, Ioannidis JP, et al (2009) The PRISMA statement for reporting systematic reviews and meta-analyses of studies that evaluate health care interventions: explanation and elaboration. J Clin Epidemiol 62: e1–34. 10.1016/j.jclinepi.2009.06.006 19631507

[46] Campbell PJ, Yachida S, Mudie LJ, Stephens PJ, Pleasance ED, Stebbings LA, et al (2010) The patterns and dynamics of genomic instability in metastatic pancreatic cancer. Nature 467: 1109–1113. 10.1038/nature09460 20981101 PMC3137369

[47] Cummings MC, Simpson PT, Reid LE, Jayanthan J, Skerman J, Song S, et al (2014) Metastatic progression of breast cancer: insights from 50 years of autopsies. J Pathol 232: 23–31. 10.1002/path.4288 24122263 PMC4288974

[48] Miao X-H, Yao Y-W, Yuan D-M, Lv Y-L, Zhan P, Lv T-F, et al (2012) Prognostic value of the ratio of ground glass opacity on computed tomography in small lung adenocarcinoma: A meta-analysis. Journal of Thoracic Disease 4: 265–271. 10.3978/j.issn.2072-1439.2012.05.09 22754665 PMC3378201

[49] Kodama K, Higashiyama M, Yokouchi H, Takami K, Kuriyama K, Mano M, et al (2001) Prognostic value of ground-glass opacity found in small lung adenocarcinoma on high-resolution CT scanning. Lung Cancer 33: 17–25. 11429192 10.1016/s0169-5002(01)00185-4

[50] Zugazagoitia J, Enguita AB, Nunez JA, Iglesias L, Ponce S (2014) The new IASLC/ATS/ERS lung adenocarcinoma classification from a clinical perspective: current concepts and future prospects. J Thorac Dis 6: S526–536. 10.3978/j.issn.2072-1439.2014.01.27 25349703 PMC4209392

[51] Sakurai H, Asamura H, Miyaoka E, Yoshino I, Fujii Y, Nakanishi Y, et al (2014) Differences in the prognosis of resected lung adenocarcinoma according to the histological subtype: a retrospective analysis of Japanese lung cancer registry data. European Journal of Cardio-Thoracic Surgery 45: 100–107. 10.1093/ejcts/ezt284 23729748

[52] Babyak MA (2004) What you see may not be what you get: a brief, nontechnical introduction to overfitting in regression-type models. Psychosom Med 66: 411–421. 15184705 10.1097/01.psy.0000127692.23278.a9

[53] Peduzzi P, Concato J, Kemper E, Holford TR, Feinstein AR (1996) A simulation study of the number of events per variable in logistic regression analysis. J Clin Epidemiol 49: 1373–1379. 8970487 10.1016/s0895-4356(96)00236-3

[54] Austin PC, Mamdani MM, Juurlink DN, Hux JE (2006) Testing multiple statistical hypotheses resulted in spurious associations: a study of astrological signs and health. Journal of clinical epidemiology 59: 964–969. 16895820 10.1016/j.jclinepi.2006.01.012

[55] Maxwell SE, Delaney HD (1993) Bivariate Median Splits and Spurious Statistical Significance. Psychological Bulletin 113: 181–190.

[56] Brooks FJ, Grigsby PW (2014) The effect of small tumor volumes on studies of intratumoral heterogeneity of tracer uptake. J Nucl Med 55: 37–42. 10.2967/jnumed.112.116715 24263086 PMC4017737

[57] Taylor JMG, Ankerst DP, Andridge RR (2008) Validation of Biomarker-Based Risk Prediction Models. Clinical Cancer Research 14: 5977–5983. 10.1158/1078-0432.CCR-07-4534 18829476 PMC3896456

[58] Michiels S, Koscielny S, Hill C (2007) Interpretation of microarray data in cancer. Br J Cancer 96: 1155–1158. 17342085 10.1038/sj.bjc.6603673 PMC2360153

[59] Kalpathy-Cramer J, Freymann JB, Kirby JS, Kinahan PE, Prior FW (2014) Quantitative Imaging Network: Data Sharing and Competitive AlgorithmValidation Leveraging The Cancer Imaging Archive. Transl Oncol 7: 147–152. 24772218 10.1593/tlo.13862 PMC3998686

[60] Galavis PE, Hollensen C, Jallow N, Paliwal B, Jeraj R (2010) Variability of textural features in FDG PET images due to different acquisition modes and reconstruction parameters. Acta Oncol 49: 1012–1016. 10.3109/0284186X.2010.498437 20831489 PMC4091820

[61] Leijenaar RT, Carvalho S, Velazquez ER, van Elmpt WJ, Parmar C, Hoekstra OS, et al (2013) Stability of FDG-PET Radiomics features: an integrated analysis of test-retest and inter-observer variability. Acta Oncol 52: 1391–1397. 10.3109/0284186X.2013.812798 24047337 PMC4533992

[62] Balagurunathan Y, Gu Y, Wang H, Kumar V, Grove O, Hawkins S, et al (2014) Reproducibility and Prognosis of Quantitative Features Extracted from CT Images. Transl Oncol 7: 72–87. 24772210 10.1593/tlo.13844 PMC3998690

[63] Brooks F (2013) On some misconceptions about tumor heterogeneity quantification. European Journal of Nuclear Medicine and Molecular Imaging 40: 1292–1294. 10.1007/s00259-013-2430-y 23632962

[64] Cheng NM, Fang YH, Yen TC (2013) The promise and limits of PET texture analysis. Ann Nucl Med 27: 867–869. 10.1007/s12149-013-0759-8 23943197

[65] Ioannidis JPA, Greenland S, Hlatky MA, Khoury MJ, Macleod MR, Moher D, et al (2014) Increasing value and reducing waste in research design, conduct, and analysis. The Lancet 383: 166–175. 10.1016/S0140-6736(13)62227-8 24411645 PMC4697939

[66] Aerts HJ, Velazquez ER, Leijenaar RT, Parmar C, Grossmann P, Cavalho S, et al (2014) Decoding tumour phenotype by noninvasive imaging using a quantitative radiomics approach. Nat Commun 5: 4006 10.1038/ncomms5006 24892406 PMC4059926

[67] Altman DG, Royston P (2000) What do we mean by validating a prognostic model? Stat Med 19: 453–473. 10694730 10.1002/(sici)1097-0258(20000229)19:4<453::aid-sim350>3.0.co;2-5

[68] Chatfield C (1995) Model Uncertainty, Data Mining and Statistical Inference. Journal of the Royal Statistical Society Series A (Statistics in Society) 158: 419–466.

