# Supplementary material for: False Discovery Rates in PET and CT Studies with Texture Features: A Systematic Review
Source: PLoS One. 2015 May 4;10(5):e0124165. doi: 10.1371/journal.pone.0124165 (PMC4418696; doi:10.1371/journal.pone.0124165)
Supplement: S1 Table — (DOCX) [file pone.0124165.s002.docx]

**Table S1** Electronic search strategy for Medline Ovid interface

| 1. textur*.mp. |
| --- |
| 2. positron emission tomography.mp. or Positron-Emission Tomography/ |
| 3. Tomography, X-Ray Computed/ or computer tomography.mp. |
| 4. cancer.mp. or Neoplasms/ |
| 5. Prognosis/ or prognostic.mp. |
| 6. predictive.mp. |
| 7. 2 or 3 |
| 8. 5 or 6 |
| 9. 1 and 4 and 7 and 8 |
| 10. limit 9 to (humans and yr="2000 -Current") |
