# Supplementary material for: False Discovery Rates in PET and CT Studies with Texture Features: A Systematic Review
Source: PLoS One. 2015 May 4;10(5):e0124165. doi: 10.1371/journal.pone.0124165 (PMC4418696; doi:10.1371/journal.pone.0124165)
Supplement: S2 Table — (DOCX) [file pone.0124165.s003.docx]

**Table 2S** Technical information of texture features implementation in CT studies.

| Author | Miles [21] | Ng  [27] | Goh [23] | Ganeshan [19] | Ganeshan [25] | Win [28] | Zhang [31] | Yip [32] | Ravanelli [29] |
| --- | --- | --- | --- | --- | --- | --- | --- | --- | --- |
|  |  |  |  |  |  |  |  |  |  |
| Software | MATLAB | TexRad | TexRad | TexRad | TexRad | TexRad | TexRad | TexRad | TexRad |
| kVp | 120 | 120 | 120 | 140 | 140 | 140 | 120 | 120 | 120 |
| mAs | 300 | 280 | 150–200 | 80 | 80 | 80 | 300 | 180-280 | 200 |
| Section collimation | NI | 5 | 3 | NI | NI | NI | NI | NI | 1.5 |
| Pixel size (mm) | NI | 0.68 | NI | 0.98 | 0.98 | NI | 0.5 | NI | NI |
| Slice thickness (mm) | 10 | NI | NI | 3.75 | 3.75 | NI | 2.5–3 | 5 | 2–5 |
| Pixel array | 512 | 512 | 512 | NI | NI | NI | 512 | 512 | 512 |
| IV contrast | Yes | Yes | Yes | No | No | Yes | Yes | Yes | Yes |
| 2D/3D | NI | NI | NI | 2D | 2D | 2D | 2D | NI | 2D |
| Abbreviations: NI, no information provided; | | | | | | | | | |
