# Supplementary material for: False Discovery Rates in PET and CT Studies with Texture Features: A Systematic Review
Source: PLoS One. 2015 May 4;10(5):e0124165. doi: 10.1371/journal.pone.0124165 (PMC4418696; doi:10.1371/journal.pone.0124165)
Supplement: S3 Table — (DOCX) [file pone.0124165.s004.docx]

**Table 3S** Technical information of texture features implementation in PET studies.

| Author | El Naqa [22] | Tixier [24] | Cook [20] | Vaidya [26] | Willaime [9] | Cheng  [30] |
| --- | --- | --- | --- | --- | --- | --- |
| Software | NI | NI | NI | In-house | Analyze | Chang-Gung Image Texture Analysis toolbox/MATLAM |
| Dose (MBq) | NI | 5/Kg | 350-400 | 555–740 | 370 | 370–555 |
| Uptake period (min) | NI | 54 | 90 | NI | 60 | 50 |
| Emission time (min) | NI | NI | NI | 2–4 | NI | NI |
| Pixel size (mm) | 5.3 | NI | 5 | 5.3 | 2.61 | 2.16–4.8 |
| Slice thickness | 3.4 | NI | 3.27 | 3.4 | 2.42 | NI |
| Pixels array | NI | NI | NI | 128 | NI | NI |
| Attenuation correction | CT | CT | CT | CT | NI | CT |
| Reconstruction algorithm | OSEM | OSEM | OSEM | OSEM | OSEM/FBP | OSEM |
| 2D/3D | 3D | 3D | 3D | 3D | 3D | 3D |
| Texture feature formulas | Haralick | Haralick/  Amadasun | Amadasun | Haralick | Haralick/  Amadasun | Haralick/  Amadasun |
| Abbreviations: NI, no information provided; OSEM, ordered-subset expectation-maximization; FBP, filtered back-projection; | | | | | | |
